# Determinants of Self-Perceived Health: The Importance of Physical Well-Being but Also of Mental Health and Cognitive Functioning

**DOI:** 10.3390/bs12120498

**Published:** 2022-12-06

**Authors:** Martina Caramenti, Isabella Castiglioni

**Affiliations:** 1 Institute of Bioimaging and Molecular Physiology, National Research Council (IBFM-CNR), Via Gaetano Previati 1/e, 23900 Lecco, Italy; 2 Department of Physics “Giuseppe Occhialini”, University of Milan-Bicocca, Piazza della Scienza 3, 20126 Milan, Italy

**Keywords:** self-perceived health, chronic diseases, mental health, cognitive function, quality of life, wellbeing, functional status

## Abstract

With life expectancy increasing for the general population, public health promotion activities should be a priority to aim at a reduction of the burden and costs of hospitalization, disability, and lifelong treatment. This study aimed to explore the influence of parameters pertaining to different aspects of well-being, including physical and mental health and cognitive functioning, on self-perceived health, a predictor of chronic disease prevalence and mortality. We used data from the Survey of Health, Aging and Retirement in Europe (SHARE) project gathered between 2013 and 2017, obtaining a sample of 96,902 participants (63.23 ± 6.77 years). We found a strong association between the self-perceived health rating and not only physical health aspects but also mental health and cognitive functioning. In particular, BMI, chronic diseases and medications, muscle strength, and mobility issues had a strong effect on self-perceived health, as also did the quality of life, depression, and verbal fluency, while other aspects, such as individual characteristics, limitations in daily activities, and pain, among others only had a small effect. These results show that public health and prevention interventions should prioritize the targeting of all aspects of well-being and not only of physical health, acknowledging self-perceived health rating as a simple tool that could help provide a complete overview of psycho-physical well-being and functional status.

## 1. Introduction

Self-perceived health (SPH) is a robust concept that defines the individual’s perception of their own health status and is a useful tool to monitor health changes [1]. It allows an overall assessment of the respondent’s health in general, but it has also been linked to prediction of mortality [2,3,4]. The reliability of this type of self-assessment has been defined as good as or even better than measures linked to chronic diseases, functional ability, and psychological well-being [5], also being predictive of aspects such as chronic disease incidence [2,6] and functional decline [6,7].

The SPH value measures self-perceived health using a single item rated on a 5-point Likert scale ranging between “excellent” and “poor”, with lower values indicating a better rated health. Studies suggest that the ratings are based on more than just physical status and that the “poor” part of the scale is predominantly related to health issues, while the opposite part of the scale offers a more complete view of health [8,9], thus not only implying the absence of health issues but considering also other possible determinants linked to fitness and general well-being [10]. This is in line with the definition of health by the World Health Organization, indicating not only a lack of disease or disability but complete physical, mental, and social well-being [11]. Studies have shown that the absence of limitations in daily activities is a great determinant of SPH [12], as are also the functional status [13], the number of chronic conditions and pain [13]. Moreover, not only has psychological well-being been linked to health perception [7], in particular self-esteem, feelings of distress, and depression [14], but also cognitive functioning [15].

Here, we analyze the impact of parameters that pertain to different aspects of human health and to the quality of life, such as physical and mental well-being and cognitive functioning on self-perceived health in a middle and older aged populations in different European countries.

## 2. Materials and Methods

### 2.1. Data

For this cross-sectional study, we used the Survey of Health, Aging and Retirement in Europe (SHARE) panel database. The design and the methodological details of the survey are described elsewhere [16,17,18,19,20,21,22,23]. SHARE is a large multidisciplinary and cross-national study that provides information on socio-demographics, physical, mental, and behavioral health, with recorded data spanning over the years 2004–2020 in 8 waves. In this study, we used waves 5 to 7, which include the surveys from 2013 to 2017, for a total of 211,637 participants. Wave 8 was excluded due to data acquisition being interrupted by the outbreak or the COVID-19 pandemic in 2020, thus because of the possible influence of lockdown and restrictions on the variables of interest. The original sample consisted of participants aged 24 to 91, including household members of the survey respondents. For this study, we restricted the sample to participants aged 50 to 75 to exclude the influence of the reduced number of participants above that age while also removing younger household members. We selected only participants from European countries according to the United Nations (UN) geoscheme for Europe [24]. After restricting the age and the countries of the participants and removing respondents with missing values, the dataset comprised 96,902 participants (63.23 ± 6.77 years), of which 53,385 (55.09%) were female, and the remaining 43,517 (44.91%) were male. Table 1 shows the participants’ characteristics.

### 2.2. Self-Perceived Health (SPH)

The SPH value measures self-perceived health using a single item rated on a 5-point Likert scale. Participants had to respond to the question “Would you say your health is…” using answer categories between “excellent” and “poor” (i.e., 1 = excellent, 2 = very good, 3 = good, 4 = fair, 5 = poor). Lower SPH values indicate a better perceived health.

### 2.3. Individual Characteristics, Physical and Mental Health, Cognitive Functioning

For the analysis, we take into account individual characteristics and different variables linked to physical and mental health and cognitive functioning. We included individual characteristics, such as sex (0 = Male, 1 = Female), age at the interview, and country of domicile. For further analysis, the UN geoscheme was used to group countries to obtain greater homogeneity as suggested by the UN Statistics Division [24]. Table 2 describes the parameters regarding mental health, physical health, and cognitive functioning that were used for analysis. The division of the parameters was based on the corresponding SHARE questionnaire modules.

For further information about the physical and mental health and cognitive functioning parameters used in this study, please refer to the SHARE Scales and Multi-Item Indicators Manual [25].

### 2.4. Statistical Analysis

Data analysis was performed using R version 4.0.3 [26]. Normality was assessed with the Shapiro–Wilk test. The method for correlation analysis was defined based on the normality results. The Welch *t*-test was used to compare means due to the unequal sample sizes. Linear regression analysis was used to assess the effect of the single variables on self-perceived health estimation. For linear regression analysis, numerical variables were normalized, rescaling the range in [0,1]. The best overall linear regression model was identified as the model that maximized the adjusted R-squared (adjusted R^2^) and minimized the prediction errors (Mallows Cp and Bayesian information criteria (BIC)) using the selected variables, confirming the selection also with a 10-fold cross-validation that identified the lowest prediction error. The significance level was set at alpha = 0.05. In the case of multiple comparisons, the alpha level was adjusted using Bonferroni correction.

## 3. Results

### 3.1. Mapping Self-Perceived Health

**Figure 1:**
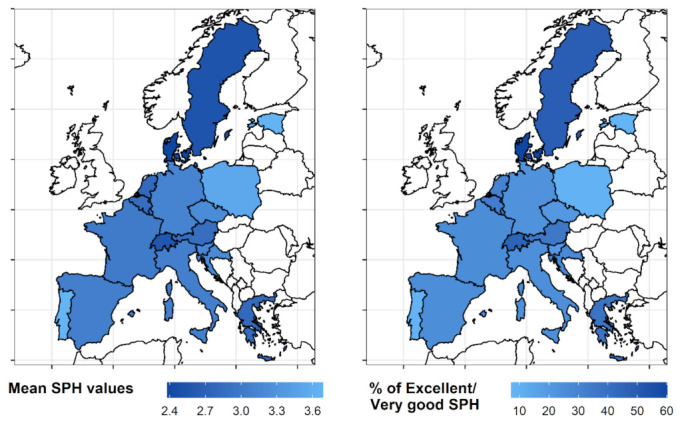
Mean Self-Perceived Health (SPH) values and prevalence of a health status rated as excellent or very good based on the country of domicile.

When using a dichotomized version of the SPH, the proportion of the selected older adult population indicating an excellent or very good health status ranges from 7.24% (Estonia) to 60.17% (Denmark) based on the country of domicile. Figure 1 shows the mean values of SPH, and the prevalence of a health status rated as excellent or very good in each country.

The proportion of individuals that rated their SPH as excellent or very good was higher in men (30.77%) than in women (29.07%). The better rating decreased with age, with a significant difference between the higher rating group (61.81 ± 6.71 years) compared to the lower rating group (63.83 ± 6.71 years). Table 3 and Table 4 present a complete overview of the distribution of the rating of SPH of the categorical variables and the mean values by SPH rating for numeric values.

For all numerical values presented in Table 4, the difference between the two groups, that is, between the higher and lower health rating, was statistically significant even after applying the Bonferroni correction (overall alpha = 0.05).

### 3.2. Linear Model

The effect of all the described parameters on the rating of SPH was assessed using a linear model comparison. For each model size up to the number of predictor variables in the data, we computed the best set of variables, selecting the model maximizing adjusted R2 and minimizing prediction errors. The best overall linear regression model was the same regardless of selection criteria and included 19 out of 20 predictor variables, only excluding ADL. This selection was also confirmed by using the 10-fold cross-validation. Due to the differences of SPH values in the different countries, we used the UN geoscheme grouping and added it as a variable to the model to see if the addition of this information could further improve the model. Since there was no increase in the adjusted R2 of the new model (adjusted R^2^ = 0.47) compared to the reduced model (adjusted R^2^ = 0.47), we used the latter for further analysis. The model was built using values normalized in a [0,1] range. Table 5 presents a complete overview of the selected model showing the effects of the variables on the estimation of SPH.

## 4. Discussion

Here, we analyzed the influence of parameters that pertain to different aspects of human health and to the quality of life on SPH rating among adults and older adults across Europe. The results highlight the impact not only of physical health but also of mental health and cognitive functioning on the estimation of one’s health status. While our results confirm that physical health has the greatest impact on SPH, they also show that aspects of mental health and cognitive function can strongly influence this rating. This is fundamental for public health promotion and prevention interventions, which should focus not only on physical factors but should adopt broader strategies, including also mental and cognitive well-being, recognizing the importance of all possible determinants.

While there are socio-economic factors that have been shown to influence the perception of one’s health, such as level of education and income [27], here, we focused on different aspects of health (physical, mental, and cognitive) and on individual characteristics (i.e., age, sex).

Studies show that men tend to see their health with a more favorable attitude compared to women [28], basing their judgment on critical illness, while women evaluate both serious and minor health problems [29]. While our results also show this tendency, the impact of sex on SPH was not as determinant as other factors, confirming the small effect found by other researchers [1]. The same also applies to the relationship between age and SPH. While there was a significant age difference between the participants that rated their health as excellent or very good and those with a poor rating, when also considering all other possible determinants, the effect was found to be small, which is in line with previous studies [1,30].

Physical health is the category that explains the vast majority of the variation of SPH, but not all aspects have large effects on the rating. While aspects such as BMI, skeletal muscle function, number of chronic diseases, and drugs, and mobility have a greater impact, there are also variables that have a minimal effect on the rating of SPH, such as pain, IADL, work limitation, and frailty.

Studies show that excessive BMI values are associated with a higher probability of low self-perceived health status, thus with greater odds of reporting poor or fair health [31,32,33,34]. This is possibly due to the great impact that excess body weight can have on multiple aspects that are associated with low self-perceived health, such as the increased risk for chronic diseases and limitations in mobility, especially for daily activities [35,36,37]. Both the presence and the number of chronic diseases have been shown to be directly associated with SPH [27,38], with the latter being defined as one of the strongest determinants of poor SPH [39,40]. Our results confirm this link but highlight that the number of chronic conditions has a greater impact on the rating of SPH compared to the presence of long-term disease. This could be due to the subjective representation of illness [41], which could influence the perception of the disease itself and the consequences, thus having a different impact on the perception of health. Moreover, our results show that there are other aspects that have a stronger correlation with SPH. The poorer rating could not only be directly linked to the perception of the health status but also to worries about one’s future and to the cost that could have to be sustained [42]. The latter could also have an impact on the strong association between the number of drugs and SPH, together with the direct influence on health perception, clearly relating to the presence of disease. Studies show that the number of medications, and in particular polypharmacy (i.e., concurrent use of 5 or more medications) [43], is associated with SPH, in particular, higher numbers of drugs are linked to poorer reported SPH [27,44]. Besides these two aspects, which clearly have an influence on the rating of one’s health, also skeletal muscle function has a great impact on SPH. This aspect is measured thanks to grip strength, a quick and simple measure to perform [45]. Our results highlight the importance of this aspect, which is in line with other studies that found that low grip strength is associated with lower odds of good ratings of SPH [46]. This could be linked to grip strength being a known indicator of physical function, with lower values being associated with possible future disability and decline [46], thus possibly influencing older individuals’ perception of health. Moreover, skeletal muscle function is also linked to some of the aspects of functional limitations, which can lead to difficulties in activities of everyday life and can be considered predictors of disability [47]. Functional limitations influence different aspects of everyday life, from mobility to fine motor limitations, also including difficulties with activities of daily living related to self-care activities, which are fundamental for independence, and instrumental activities, which are more linked to independent life in the community [47]. While studies show an important relationship between SPH and all aspects of functional limitations [46,48,49], here we found that only some of the aspects had a great impact on the rating of SPH. In fact, ADL did not show a significant effect on SPH after adjusting for all variables, so it was excluded from the model analyzed in this study. Studies hypothesize that this may be due to the fact that SPH may be able to capture aspects of health that are not fully exposed by ADL [50]. While having a significant effect on the rating of SPH, also GALI and IADL did not have a great impact based on our results. The effect of GALI, which is used as the underlying measure of disability-free life expectancy in European surveys, was greater compared to IADL, probably due to the fact that ADL and IADL cover only a fraction of possible activities, but on the other hand, the density of concepts that are condensed into one single question for the GALI (“For the past 6 months at least, to what extent have you been limited because of a health problem in activities people usually do?”) may have nevertheless influenced its impact [51]. Moreover, studies show that older people may have a tendency to report fewer functional limitations underestimating difficulties due to adjustments with respect to the aging process, thus accepting some decline due to advancing age [52]. A greater effect was found for the mobility variable, which included not only mobility but also arm function and fine motor limitations. These limitations heavily influence the degree of dependence and may have a great impact on participation in social and cultural activities [53], decreasing quality of life [54]. Moreover, studies show that as the degree of dependence increases, this may be perceived as a weakness, likely negatively influencing the rating of SPH [55]. These limitations could have also influenced the effect of limitations in paid work on SPH. In fact, while studies show that poor health is directly linked to exit from paid work [56], our results show that limitations in paid work only have a small effect on the perception of one’s health. This could be due to the development of policies that encourage the delay of retirement or that require older workers to remain longer in employment [57] so that even a possible mismatch between the requirements of the job and the individual’s capacities may be perceived as normal due to aging, thus reducing the impact on the perception of health itself. This could also explain the reduced effect of pain and frailty on SPH due to the possible influence of what is perceived as the normal aging process [58,59,60].

While the physical health category is the one that has the greatest effect overall on SPH rating, explaining a high proportion of the variance which is captured by the complete model, there are also aspects of mental health and cognitive functioning that can be of interest due to their impact on SPH. In particular, our results show the great impact on SPH rating of the CASP scale, an indicator of the quality of life measuring control, autonomy, self-realization, and pleasure, while the effect was reduced for depression (measured by the EURO-D scale) and extremely low for loneliness. Studies have shown the direct association between SPH and the quality of life, with the perception of one’s own health influencing lifestyle choices, which in turn can affect the quality of life [61]. This association shows the impact of different aspects of mental health on the perception of health status, even if the latter is more associated with physical functioning. This is also true for depressive symptoms, even if studies do not agree on the causality mechanisms underlying the well-established connection between SPH and depression [14]. Researchers hypothesize that mental health issues such as depression may function as stressors with a potentially negative effect on physical health [62,63,64]. Loneliness, on the other hand, did not have a great effect on SPH, even if this correlation has been shown in previous studies [65]. This could be due to the age of our sample, since some researchers found the strongest association between the age of 30 and 59, while for older participants, it was lower, even if still significant [66]. Besides these mental health aspects, also cognitive functioning has an impact on the rating of SPH. Studies show that low SPH ratings are associated with impaired cognitive abilities [15], but our results highlight this association only for verbal fluency, while there is only a smaller effect of recall, both immediate and delayed. Previous studies confirm the influence of the cognitive domain on SPH rating [61], but the results are not consistent [61,67]. This could be linked to the fact that some people may not even consider their health to be affected by cognitive impairment in general [68], especially for milder cases, but limitations in communication due to poor verbal fluency could be more noticeable.

Future work should concentrate on analyzing the impact of physical, mental, and cognitive aspects on self-perceived health using longitudinal data due to the inherent limitation of cross-sectional studies that do not allow to infer causation.

## 5. Conclusions

Due to the increased life expectancy of the general population, public health promotion activities should be a priority for all countries aiming at reducing the burden of hospitalization, disability, and the need for lifelong treatment that derive especially from the rise of chronic diseases. Public health and prevention interventions should prioritize the targeting of all aspects of health, including physical and mental health and cognitive functioning, also acknowledging the usefulness of simple self-assessment methods, such as SPH, that could allow for simple monitoring of health and prediction of mortality. This could help detect aspects of health able to postpone the onset of disease, thus promoting healthy aging.

## Figures and Tables

**Table 1:** Participants’ characteristics by country and sex.

UNGeo	Country	Total Participants	Male Participants				Female Participants			
			*n*	%	Mean Age	SD Age	*n*	%	Mean Age	SD Age
**Eastern** **Europe**	Czech Rep.	7594	3147	41.44	64.55	6.12	4447	58.56	64.55	6.12
	Poland	2038	885	43.42	64.09	6.09	1153	56.58	64.09	6.09
**Northern Europe**	Denmark	6917	3260	47.13	62.72	6.88	3657	52.87	62.72	6.88
	Estonia	6972	2716	38.96	63.21	6.84	4256	61.04	63.21	6.84
	Sweden	6409	2953	46.08	65.53	6.10	3456	53.92	65.53	6.10
**Southern Europe**	Croatia	1876	861	45.9	62.65	6.53	1015	54.10	62.65	6.53
	Greece	4591	2009	43.76	64.42	6.50	2582	56.24	64.42	6.50
	Italy	7633	3452	45.22	64.04	6.96	4181	54.78	64.04	6.96
	Portugal	1109	491	44.27	64.61	5.93	618	55.73	64.61	5.93
	Slovenia	4945	2180	44.08	63.04	6.44	2765	55.92	63.04	6.44
	Spain	7729	3661	47.37	63.82	6.48	4068	52.63	63.82	6.48
**Western Europe**	Austria	5460	2309	42.29	64.48	6.56	3151	57.71	64.48	6.56
	Belgium	8951	4142	46.27	62.34	6.83	4809	53.73	62.34	6.83
	France	6487	2920	45.01	63.18	6.37	3567	54.99	63.18	6.37
	Germany	8078	3846	47.61	62.98	7.25	4232	52.39	62.98	7.25
	Luxembourg	2333	1118	47.92	62.17	6.56	1215	52.08	62.17	6.56
	Netherlands	3090	1414	45.76	63.03	6.56	1676	54.24	63.03	6.56
	Switzerland	4690	2153	45.91	64.33	6.36	2537	54.09	64.33	6.36

**Table 2:** Description of the physical and mental health and cognitive functioning parameters used in this study.

Category	Parameter	Scale	Description
**Mental health**	EURO-D scale	Numeric [1,12] 1 = Not depressed 12 = very depressed	12-item depression scale
	CASP-12	Numeric [12,48] 12 = Low quality of life 48 = High quality of life	Revised 12-item version of the 19-item CASP-19 for the measure of the quality of life
	Loneliness	Numeric [3,9] 3 = Not lonely 9 = Very lonely	3-item loneliness scale based on the R-UCLA Loneliness Scale
**Physical health**	GALI	Categorical 1 = Severely limited 2= Limited, but not severely 3 = Not limited	Global Activity Limitation Index—indicator measuring long-standing activity limitations linked to general health problems
	ADL	Numeric [0,6] 0 = No difficulties 6 = Higher limitations	Activities of Daily Living—number of limitations with everyday self-care activities referring to the maintenance of independence
	IADL	Numeric [0,7] 0 = No difficulties 7 = Higher limitations	Instrumental Activities of Daily Living—Modified 7-item index describing the number of limitations with instrumental activities of everyday life
	Pain	Categorical 0 = Yes 1 = No	Single value indicating if the participant is troubled with pain.
	BMI	Numeric	Body Mass Index—measure for evaluating body weight in relation to height (kg/m^2^)
	Maximum grip strength	Numeric	Maximum value of the grip measurements of both hands (kg). It is considered an indicator of skeletal muscle function
	Number of chronic diseases	Numeric	Number of chronic diseases
	Mobility	Numeric [0,10] 0 = No difficulties 10 = Higher limitations	Mobility, arm function, and fine motor limitations
	Number of drugs	Numeric	Number of drugs taken at least once a week
	Long term illness	Categorical 0 = Yes 1 = No	Presence of long-term illness
	Work limitation	Categorical 0 = Yes 1 = No	Health problems that limit paid work
	Frailty	Categorical 0 = Yes 1 = No	Bothered by frailty
**Cognitive functioning**	Immediate recall	Numeric [0,10]	Number of words that the respondent is able to recall immediately after the encoding phase during the 10-word recall test
	Delayed recall	Numeric [0,10]	Number of words that the respondent is able to recall after a delay time during the 10-word recall test
	Verbal fluency	Numeric [0,100]	Number of correct words from a semantic category that the respondent says in 60 s

**Table 3:** Overview of the distribution of the rating of categorical variables.

Variables	Excellent/Very Good SPH	%	Total
**Sex**			
Male	13,389	30.77	43,517 (44.91%)
Female	15,521	29.07	53,385 (55.09%)
**GALI** ^1^			
Not limited	25,582	43.22	59,193 (61.09%)
Limited	3328	8.83	37,709 (38.91%)
**Pain**			
Yes	5144	13.01	39,541 (40.81%)
No	23,766	41.43	57,361 (59.19%)
**Long-term illness**			
Yes	5742	12.71	45,179 (46.62%)
No	23,168	44.79	51,723 (53.38%)
**Work limitation**			
Yes	1290	6.57	19,640 (20.27%)
No	27,620	35.75	77,262 (79.73%)
**Frailty**			
Not selected	3342	11.64	28,718 (29.64%)
Selected	25,568	37.5	68,184 (70.36%)

^1^ Global Activity Limitation Index.

**Table 4:** Overview of mean values by self-perceived health rating.

	Very Good		Less than Very Good	
	Mean	SD	Mean	SD
**Age**	61.81	6.71	63.83	6.71
**EURO-D ^1^**	1.35	1.55	2.53	2.22
**CASP ^2^**	41.13	4.82	37.13	5.99
**Loneliness**	3.45	0.93	3.87	1.33
**ADL ^3^**	0.02	0.19	0.16	0.6
**IADL ^4^**	0.03	0.26	0.23	0.78
**BMI ^5^**	25.79	3.86	27.57	4.83
**Maximum grip strength**	37.54	11.29	34.32	11.53
**Number of chronic diseases**	0.79	0.97	1.9	1.52
**Mobility**	0.28	0.77	1.51	2.06
**Drugs**	0.79	0.99	1.94	1.64
**Immediate recall**	6.07	1.55	5.48	1.62
**Delayed recall**	4.91	1.97	4.12	2.07
**Verbal fluency**	23.49	7.68	20.79	7.38

^1^ Depression scale. ^2^ Quality of life scale. ^3^ Activities of Daily Living. ^4^ Instrumental Activities of Daily Living. ^5^ Body Mass Index.

**Table 5:** Self-Perceived Health estimation: Overview of the model coefficients by category. Note. Adjusted R^2^ = 0.47. CI = Confidence Interval for B.

Category	Parameter	B (SE)	95% CI	*p*
	(Intercept)	0.784 (0.007)	[0.77 0.8]	<0.001
**Individual characteristics**	Age	0.02 (0.002)	[0.02 0.03]	<0.001
	Sex	−0.036 (0.002)	[−0.04 −0.03]	<0.001
**Mental health**	EURO-D scale ^1^	0.102 (0.004)	[0.09 0.11]	<0.001
	CASP-12 ^2^	−0.248 (0.005)	[−0.26 −0.24]	<0.001
	Loneliness	−0.022 (0.003)	[−0.03 −0.02]	<0.001
**Physical health**	GALI ^3^	0.08 (0.002)	[0.08 0.08]	<0.001
	IADL ^4^	−0.024 (0.01)	[−0.04 −0.01]	0.011
	Pain	−0.04 (0.001)	[−0.04 −0.04]	<0.001
	BMI ^5^	0.262 (0.012)	[0.24 0.29]	<0.001
	Maximum grip strength	−0.131 (0.009)	[−0.15 −0.11]	<0.001
	Number of chronic diseases	0.191 (0.008)	[0.17 0.21]	<0.001
	Mobility	0.121 (0.005)	[0.11 0.13]	<0.001
	Number of drugs	0.133 (0.007)	[0.12 0.15]	<0.001
	Long term illness	−0.073 (0.002)	[−0.08 −0.07]	<0.001
	Work limitation	−0.046 (0.002)	[−0.05 −0.04]	<0.001
	Frailty	−0.031 (0.002)	[−0.03 −0.03]	<0.001
**Cognitive functioning**	Immediate recall	−0.03 (0.005)	[−0.04 −0.02]	<0.001
	Delayed recall	−0.066 (0.004)	[−0.07 −0.06]	<0.001
	Verbal fluency	−0.129 (0.009)	[−0.15 −0.11]	<0.001

^1^ Depression scale. ^2^ Quality of life scale. ^3^ Global Activity Limitation Index. ^4^ Instrumental Activities of Daily Living. ^5^ Body Mass Index.

## Data Availability

This paper uses data from SHARE Waves 5, 6 and 7 (DOIs: 10.6103/SHARE.w5.710, 10.6103/SHARE.w6.710, 10.6103/SHARE.w7.710).
